# Cutting-edge knowledge on the roles of phytobiotics and their proposed modes of action in swine

**DOI:** 10.3389/fvets.2023.1265689

**Published:** 2023-09-20

**Authors:** Sriniwas Pandey, Eun Sol Kim, Jin Ho Cho, Minho Song, Hyunok Doo, Sheena Kim, Gi Beom Keum, Jinok Kwak, Sumin Ryu, Yejin Choi, Juyoun Kang, Jeehwan Choe, Hyeun Bum Kim

**Affiliations:** ^1^Department of Animal Resources Science, Dankook University, Cheonan, Republic of Korea; ^2^Division of Food and Animal Science, Chungbuk National University, Cheongju, Republic of Korea; ^3^Division of Animal and Dairy Science, Chungnam National University, Daejeon, Republic of Korea; ^4^Major of Beef Science, Department of Livestock, Korea National University of Agriculture and Fisheries, Jeonju, Republic of Korea

**Keywords:** phytobiotics, swine, health, growth performance, modes of action

## Abstract

With the ban on antibiotics in the swine industry, the exploration of alternative options has highlighted phytobiotics as a promising substitute for antibiotic growth promoters, aiming to foster a more sustainable swine industry. Phytobiotics are non-nutritive natural bioactive components derived from plants that offer numerous health benefits. They exhibit antioxidative, antimicrobial, and anti-inflammatory effects. Phytobiotics can be utilized in various forms, including solid, dried, ground, or as extracts, either in crude or concentrated form. They are characterized by low residual levels, a lack of resistance development, and minimal adverse effects. These qualities make phytobiotics an attractive choice for enhancing health and productivity in swine, presenting them as a viable alternative to antibiotics. While there is a general understanding of the effects of phytobiotics, there is still a need for detailed information regarding their effectiveness and mechanisms of action in practical settings. Therefore, the purpose of this mini review was to summarize the current knowledge supporting the roles of phytobiotics and their proposed modes of action, with a specific focus on swine.

## Introduction

The growing concerns over the use of antibiotics as growth promoters in livestock feed have led to a search for better alternatives that can provide similar effects and performance without causing severe negative impacts. In response to this, numerous substances have been studied and found to possess good qualities that aid and improve the health and overall growth of livestock. These substances play major roles in maintaining normal physiological functions and animal health, as well as protecting animals from infectious diseases. One such substance that has been identified is termed “nutraceutical” (1). Nutraceuticals refer to substances that are produced in a purified or extracted form and administered to animals with the purpose of improving their health and well-being (2). This group includes various types of substances such as enzymes, synbiotics, organic acids, polyunsaturated fatty acids, and phytobiotics (3, 4).

Phytobiotics, a specific type of nutraceuticals, are non-nutritive plant-derived natural bioactive components that can be used as feed additives (5–8). These phytobiotics have been extensively studied for their ability to improve the overall growth performance and health of animals. Notably, phytobiotics possess several desirable attributes, including low residue levels, absence of resistance development, and minimal side effects (9–11). These qualities make them a promising option for promoting animal health and productivity in a sustainable and responsible manner (7, 12–14). To date, over 5,000 different dietary phytobiotics have been discovered from a variety of sources including fruits, vegetables, legumes, whole grains, herbs, and essential oils. It is widely accepted that phytobiotics can be used in various forms, such as solid, dried, and ground or as extracts, either in crude or concentrated form, in which the accumulation of biologically active substances is greatest (15, 16). In general, phytobiotics contain essential nutrients, such as carbohydrates, along with other secondary components, including essential oils and phenolic compounds. Because phytobiotics do not significantly contribute to the intake of primary nutrients in animals, the main focus is on the secondary plant components as the main ingredients of interest in phytobiotics, even though certain polysaccharides can also act as phytobiotics (6, 17, 18). Although there are no definitive classification criteria, phytobiotics can be classified into 4 different categories based on their origin and processing characteristics: (1) herbs (blooming, nonwoody, and nonpersistent plants); (2) spices (plants with a strong odor or flavor); (3) essential oils (volatile lipophilic components); and (4) oleoresins (extracts derived from non-aqueous solutions) (Table 1) (13, 25).

**Table 1:** Classification of phytobiotics and their functional examples addressed in this mini review.

Classification	Types	Functions	References
Herbs	Bloomin Nonwoody Nonpersistent plants	Improve intestinal microflora	Deng et al. (19) Li et al. (9)
		Antimicrobial effects	Wang et al. (20)
		Antioxidative and anti-inflammatory effects	Weber et al. (21)
			Esatbeyoglu et al. (22)
			Spanier et al. (23)
			Wei and Shibamoto (24)
			Gheisar and Kim (25)
			Filazi et al. (3)
		Growth enhancement	Davila-Ramirez et al. (26)
Spices	Plants with a strong odor Flavor that are commonly added to foods	Growth enhancement	Janz et al. (27) Al-Kassie (28)
Essential oils	Volatile lipophilic components	Enhance intestinal barrier functions	Su et al. (29) Liu et al. (30)
		Antimicrobial effects	Ahmed et al. (31)
		Growth enhancement	Li et al. (32) Manzanil et al. (33)
Oleoresins	Extracts derived from non-aqueous solutions	Improve intestinal microflora	Qu et al. (34) Satora et al. (35)
		Antimicrobial effects	Vasconcelos et al. (36)
			Girard et al. (37)
			Fu et al. (38)
		Growth enhancement	Yang et al. (39)
			Davila-Ramirez et al. (26)
			Yan et al. (40)
			Marcin et al. (41)

A variety of literature has proven the positive effects of phytobiotics, which include several beneficial outcomes. These effects encompass enhanced growth of beneficial microbes in the gut, as well as antioxidative, antimicrobial, and anti-inflammatory properties (3, 42). These functional activities of phytobiotics have been attributed to various bioactive compounds present in them. These bioactive compounds include terpenoids (mono- and sesquiterpenes, steroids), flavonoids, alkaloids (in the form of alcohols, aldehydes, ketones, esters, and lactones), phenols (tannins), glycosides and glucosinolates (17, 43, 44). However, the mechanism action of phytobiotics have not been clearly elucidated due to the wide variety of bioactive substances present in these plant-derived products. The content and chemical composition of active substances in phytobiotics can vary based on factors such as the plant part used (seeds, leaves, etc.), geographical location, and harvesting season. These variations contribute to the complexity of understanding the precise mechanisms by which phytobiotics exert their effects (13, 25, 45).

Therefore, the purpose of this mini review was to provide a summary of the current knowledge regarding the roles of phytobiotics and their proposed modes of action, particularly in swine. Despite the complexity of understanding the precise mechanisms of action due to the diverse bioactive substances and variations in content and composition, the review aimed to consolidate the existing literature and shed light on the potential benefits of phytobiotics in swine production.

### Effects of phytobiotics on the swine gut microbiome

The intestinal health of animals is crucial for their overall health and well-being, and it is associated with several aspects, including gut microbiome and mucosal barrier. Disturbances in these elements can impact animal health. Phytobiotics tend to promote the intestinal health in animals by enhancing the functions of these elements (46).

**Figure 1:**
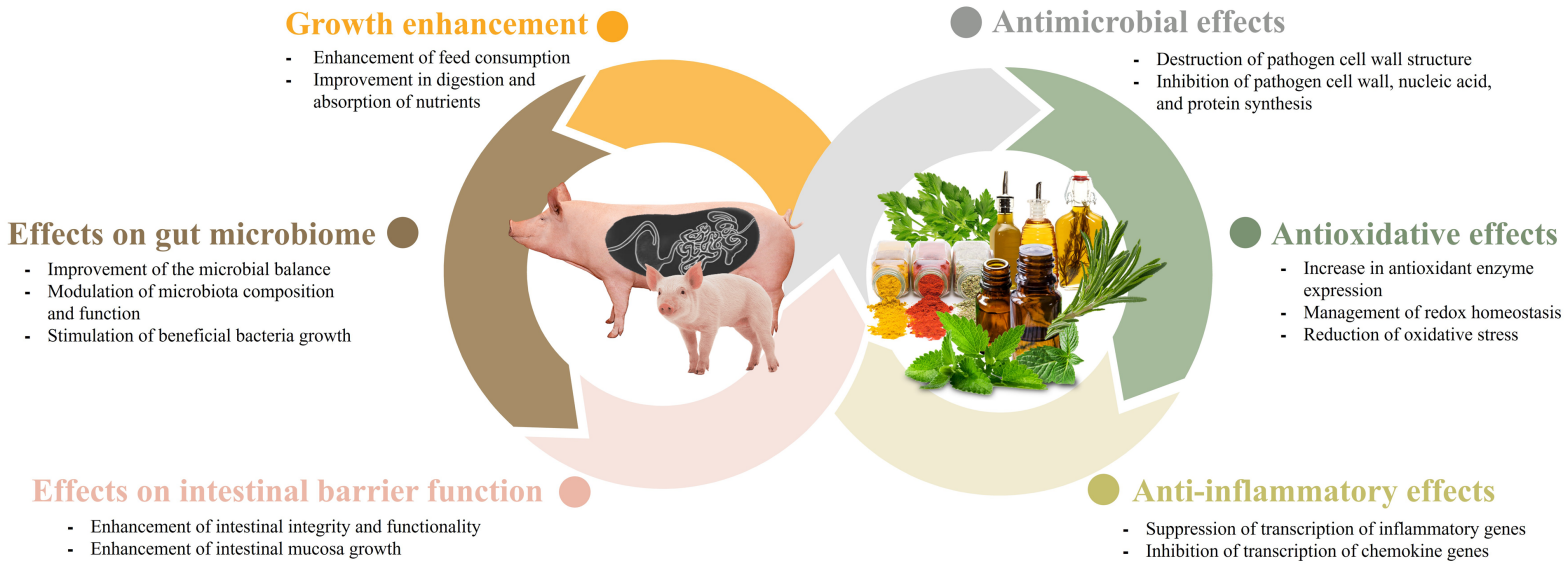
The main effects of phytobiotics in swine production systems.

The impact of phytobiotics on the gut microbiome has been extensively studied because of their significant roles in the health and productivity of livestock. Phytobiotics have been found to not only alter bacterial proliferation but also influence the composition and function of the microbiota (47). Weaned pigs experience stressors associated with changes in their feed and environment when they are moved from the farrowing room to the nursery facility. These changes can hinder the establishment of a stable gut environment. Phytobiotics have the potential to enhance the microbial balance in young pigs, resulting in improved health and feed efficiency. The presence of beneficial gut bacteria during the weaning period is crucial, and plant-based products can serve as effective means to modulate it (9, 19, 35, 45). In a study by Deng et al. (19), polysaccharides derived from cassiae seeds have been found to improve the intestinal microflora of piglets. In another study by Li et al. (48), when weaned pigs were fed a diet supplemented with coix seed, there was a significant increase in the populations of *Lactobacillus* and *Bacteroides* in the gastrointestinal tract. Additionally, there was a reduction in the abundance of *Prevotellaceae*. These findings suggest that the inclusion of coix seed in the feed can positively influence the composition of the gut microbiota in weaned pigs, promoting a healthier microbial balance. Coix seed contains 60% starch, similar to cereals, but the content of oil, polysaccharides and protein is higher than cereals, making it an enriched medium for gut microbiota. Also, proteins and polysaccharides help regulate water transportation, providing the gut microbiota with a suitable environment to grow (48). In addition, several studies have suggested that gut microbes play a role in metabolizing ingested phytobiotics into simpler metabolites. This microbial metabolism increases the bioavailability of the phytobiotics, leading to enhanced health-promoting effects in the intestine. The gut microbiota’s ability to metabolize phytobiotics into bioactive compounds highlights the intricate relationship between the host, microbiota, dietary components, and underscores the importance of considering microbial metabolism in understanding the beneficial effects of phytobiotics on intestinal health (49). In a study conducted by Fresno Rueda et al. (45), the effects of phytobiotics containing polyphenols on weaned pigs were investigated. The study found an increase in the abundance of both lactate-producers and lactate-utilizers in the gut of pigs. Lactate is an important metabolite in the gut, as it can suppress the growth of pathogens and can also be utilized by the host in the form of propionate. Furthermore, beyond the weaning period, phytobiotics continue to play a role in modulating the gut microbiota in growing-finishing pigs. For instance, supplementation of 1.5% bamboo vinegar powder was found to increase the abundance of *Firmicutes* and *Bacteroidetes*, two dominant bacterial phyla in the gut. This supplementation also promoted the richness of *Lactobacillus*, a beneficial genus, and *Thalassospira* (34). These findings highlight the potential of phytobiotics in shaping the gut microbiota composition and promoting the growth of beneficial bacteria in weaned and growing-finishing pigs. For sows with garlic supplementation, a study by Satora et al. (35) observed that the diversity and richness of the microbial community changed across the different taxonomic levels of identification. The study found an increase in species-level diversity and richness, while the trend was opposite at the family and genus levels. These findings support the idea that phytobiotics may play a role in shaping the gut microbiome (Figure 1).

### Effects of phytobiotics on the swine intestinal barrier function

Phytobiotics also play a role in maintaining intestinal barrier function (Figure 1). The integrity of the intestinal barrier depends on the interplay between various components, including the adhesive mucous gel layer, immunoglobulin A, antibacterial peptides, and intercellular tight junctions. Among these components, tight junctions are the primary factors that influence the integrity of the intestinal barrier. Tight junctions consist of transmembrane proteins, such as claudin, occludin, tricellulin, junctional adhesion molecule-A (JAM-A), as well as intracellular plaque proteins like zonula occludens (ZO) (50). The addition of resveratrol has been documented to effectively restore the expression of ZO-2, occludin, JAM-A, and claudin, while also alleviating the elevation of plasma lipopolysaccharide (LPS)-binding protein levels. Elevated levels of LPS-binding protein serve as an indicator of compromised intestinal barrier function in the animal (51). In addition, naringenin, a flavanone found in citrus fruits, has been shown to increase the expression of occludin, JAM-A, and claudin-3 (52). Also, it has been known that plant extraction oil (PEO) enhances intestinal mucosa growth in weaned pigs, attributing to the mediation of improved intestinal integrity and functions (29). In another study by Liu et al. (30), administering capsicum oleoresin (CAP) and garlic botanical (GAR) was found to increase the expression of genes related to membrane integrity in infected weaning pigs. This administration also promoted gut mucosa health, leading to improvements in diarrhea and clinical immune responses in infected pigs that were given the plant extract. Altogether, it has been shown that phytobiotics tend to promote the intestinal health of the animal by maintaining the intestinal barrier function.

### Antimicrobial effects of phytobiotics in swine

The antimicrobial effects of phytobiotics have been the subject of numerous studies and have consequently been extensively investigated (53–55).The majority of these studies have shown that phenolic components, such as thymol, carvacrol, limonene, geraniol, phenylpropane, and citronellal, are among the most potent antimicrobials (3). When it comes to the mechanisms of action, the variation in the composition of phytobiotic components has been found to significantly impact the way they act (56, 57). Nevertheless, the antimicrobial effects of phytochemical feed additives, in general, can be attributed to four different modes of action that are: (1) destruction of cell wall structure; (2) inhibition of cell wall synthesis; (3) inhibition of nucleic acid synthesis, and (4) interfering with protein synthesis (58). According to Wang et al. (20), an overview of antimicrobial mechanisms of ginseng revealed several modes of action. These include disrupting biofilm formation, destroying mature biofilms, altering lipid bilayers, and creating pores in the bacterial cell wall membrane. In addition to the mentioned antimicrobial mechanisms of ginseng, it has also been found to inhibit the efflux of antibiotics, which reduces the likelihood of drug resistance development in microbes. Furthermore, cinnamon extracts have been documented to exhibit antimicrobial activity through various mechanisms, including the disruption of cell membrane, inhibition of ATPases, interference with cell division, and inhibition of biofilm formation (36). Several studies also have reported that thymol possesses the ability to integrate into the polar-head group region of the lipid bilayer. This integration leads to the modification of the structural integrity and fluidity of the membrane through hydrogen bonding and hydrophobic interactions (59, 60).

When phytobiotics are used in swine, their antimicrobial effects have been proven to yield positive results. Essential oils have demonstrated effective antimicrobial activities when utilized in conjunction with organic acids (61). Zhou et al. (61) reported that the combination of essential oils (such as carvacrol or thymol) with acetic acid or citric acid exhibited better efficacy against *Salmonella typhimurium* compared with using individual essential oils or organic acids alone. Another study by Ahmed et al. (31) reported a decrease in the count of fecal *Salmonella* and *Escherichia coli* (*E. coli*), as well as a reduction in diarrhea scores, when benzoic acid and thymol were fed to the swine. The mechanism behind this synergism is still unclear. However, it is well-documented that phenols present in essential oil can alter the structure and functions of the bacterial cell membrane. This alteration leads to swelling, resulting in increased membrane permeability, and consequently, heightened susceptibility of bacteria to organic acids (62). Enterotoxigenic *E. coli* is considered as one of the primary causes of diarrhea in weaning piglets. A study by Girard et al. (37) showed promising result on supplementation of 2% chestnut extract (CE) immediately after weaning instead of antibiotics, improving growth performance, and reducing the occurrence of post-weaning diarrhea (PWD) caused by Enterotoxigenic *E. coli* (ETEC F4). In another study, Chang et al. (63) conducted a study to explore the effects of different combinations of phytogenic feed additives (PFA) on pigs infected with *E. coli.* Among the numerous combinations studied, the mixture of bitter citrus extract, thymol, and carvacrol demonstrated the most effective results in terms of enhancing immune response, intestinal morphology, and tight junction expression. In a study by Fu et al. (38), administration of baicalin-aluminum complex (BBA) altered the composition of the gut microbiome, leading to a significant reduction in the frequency of diarrhea in piglets. Additionally, supplementation with *Scutellaria baicalenis* extracts (SBE) has been demonstrated to effectively reduce the incidence of diarrhea in weaning piglets and decrease the expression of inflammatory cytokines by inhibiting the NF-kB and P38 signaling pathways (64). Overall, these studies demonstrate that phytobiotics can be utilized as potential alternatives to antimicrobial agents in swine (Figure 1).

### Antioxidative and anti-inflammatory effects of phytobiotics in swine

The antioxidant and anti-inflammatory properties of phytobiotics are indeed noteworthy and have garnered significant interest (Figure 1). The swine industry experiences substantial economic losses each year due to various types of oxidative stress. Phytobiotics can help mitigate these losses by providing antioxidant support and reducing inflammation in swine (65). Oxidative stress refers to a significant increase in the production of free radicals, such as reactive oxygen species (ROS) and reactive nitrogen radicals (RNS), within the bodies of animals. It can also occur when the capacity to effectively eliminate free radicals is reduced, disrupting the balance between antioxidation and oxidation processes in the body (66). Studies have identified five primary factors that can trigger oxidative stress in swine. These factors include birth, weaning stress, feeding environment, mycotoxin presence in feed, and social factors (66). Given the detrimental effects of oxidative stress, it is crucial for the swine industry to combat this condition. The unregulated production of free radicals, such as ROS and RNS, associated with oxidative stress, can even trigger inflammation (67, 68).

The antioxidant and anti-inflammatory mechanisms exhibited by phytobiotics are largely attributed to their regulation of signaling pathways, as highlighted by Li et al. (9). The antioxidant responsive element (Nrf2-ARE) signaling pathway plays a vital role in cellular response to oxidative stress (69). The transcription factor Nrf-2 binds with the antioxidant response element (ARE) and initiates the activation of a diverse range of genes, including antioxidant enzymes and proteins, that provides cellular protection against oxidative stress (70). Phytobiotics, thus help activate Nrf-2 pathway, upregulating antioxidant enzymes and several protective proteins. A number of *in vitro* and *in vivo* experiments have indicated that oxidative stress can also trigger activation of Nuclear Factor Kappa B (NF-κB) pathway. Increased expression level of NF-κB can facilitate transcription of multitude of inflammatory genes (71). This signaling pathway serves as a principal regulator of inflammation (72). NF-κB is a transcriptional factor that plays an important role in many critical physiological responses, including the inflammatory response (73). This pathway is responsible for expression of genes encoding many pro-inflammatory cytokines and chemokines (74). NF-κB activation leads to tissue alternations indicative of inflammation (9). In relation to the above mentioned information, the findings of Wang et al. (72) showed that traditional Chinese medicine (TCM) I and II, comprised of several kinds of plant derivatives, can improve antioxidative and anti-inflammatory capabilities in liver of piglets *via* activation of the Nrf2 pathway. In addition, curcumin, a compound presented in turmeric, has been shown to have anti-inflammatory and antioxidant activities, capable of scavenging free radicals and facilitating antioxidant functions through the Nrf2 signaling pathway (21, 22). Another compound, resveratrol, obtained from grapes and wine, was also found to lessen inflammation, and regulate redox homeostasis (23). In cells, redox homeostasis is the balance between generation and elimination of ROS/RNS (75). A study by Cao et al. (76) showed that resveratrol was effective in improving the redox status, decreasing mitochondrial damage, and promoting mitophagy in piglets injected with diquat. Plant species such as coriander, ginger, curcuma as well as those rich in flavonoids (in green tea) and anthocyanins (in various fruits) have antioxidant activities (24, 25). Studies showed black pepper, red pepper and chilli also possess antioxidant properties, however, most of these plants are restricted from being added specially in swine feed because of the pungent smell and taste of the active substances of these plants (3).

### Growth enhancement by phytobiotics in swine

With the ban of antibiotic growth promoters (AGPs), phytobiotics have emerged as promising alternative feed additives, as noted by Valenzuela-Grijalva et al. (58). Various studies have demonstrated the potential growth-promoting effects of phytobiotics, although the precise mechanisms underlying their role as growth enhancers have not been fully elucidated (Figure 1). However, it has been suggested by Valenzuela-Grijalva et al. (58) that phytobiotics may promote growth through several different ways. These includes: (1) through improvement of feed status and feed consumption by increasing flavor and palatability; (2) enhancement in nutrition digestion and absorption; and (3) promotes anabolic activity comparable to that of anabolic substances.

Some phytobiotics, when added to feeds, have been shown to enhance flavor and palatability, thereby increasing feed intake in swine. This improvement in feed intake can ultimately lead to improved production performance (28, 77). Janz et al. (27) discovered that pigs demonstrated a preference for feed supplemented with garlic or rosemary compared to feed supplemented with oregano or ginger. However, other studies have indicated that the palatability of feed was negatively affected when pigs were fed essential oils extracted from fennel and caraway, or from thyme or oregano (78–80).

Phytobiotics improve nutrient digestion and absorption by stimulating the secretion of various digestive secretions, including saliva, mucus, digestive enzymes, and bile. These enhanced secretions contribute to the breakdown and assimilation of nutrients from the diet. Furthermore, phytobiotics have been shown to exhibit prebiotic activity, promoting the growth and activity of beneficial gut bacteria, which further supports efficient nutrient digestion and absorption (7, 81, 82). Janz et al. (27) and Manzanil et al. (33) conducted studies where they observed a stimulating effect on the pancreatic enzymes, specifically amylase and trypsin activity, in pigs after the administration of cinnamaldehyde and an essential oil blend. These findings suggest that these phytobiotics can enhance the activity of pancreatic enzymes involved in nutrient digestion, contributing to improved nutrient utilization in pigs (25, 33, 83, 84). The increase in activity of digestive enzymes facilitates an increment in the gastric retention time of ingested feed. This prolonged retention time allows for improved digestibility and availability of nutrients. As a result, the enhanced enzymatic activity induced by phytobiotics contributes to more efficient nutrient digestion and utilization in animals (81). Li et al. (32) conducted a study on pigs fed diets supplemented with essential oils and observed significant improvements in weight gain, as well as digestibility of dry matter and crude protein. They proposed that the enhancing intestinal morphology resulting from essential oil supplementation contributed to improved nutritional digestibility, ultimately leading to better performance in pigs. Similarly, in a study by Yang et al. (39), piglets supplemented with rosemary extract (RE) exhibited increased villus height and villus height/crypt depth ratio in both the jejunum and ileum. These changes in intestinal morphology have the potential to positively impact growth performance, nutrient digestibility, and overall intestinal health in weaned piglets (32, 39).

In a study by Davila-Ramirez et al. (26), the addition of plant extracts (artichoke, beet, celery, garlic, avocado, spinach, oats, and parsley) to the diet of pigs resulted in improved average daily gain (ADG), average daily feed intake (ADFI), and final live weight (LW) under heat stress conditions during the growing to finishing period. Similarly, in a study by Yan et al. (40), supplementation with an herbal extract mixture (HEM) containing buckwheat, thyme, curcuma, black pepper, and ginger resulted in improved growth performance, specifically increased ADG and ADFI, in growing pigs compared to a non-supplemented control (NC) treatment. However, no significant changes in feed conversion ratio (FCR) were observed. Marcin et al. (41) observed a significant improvement in ADG in piglets that were administered a diet supplemented with extracts of sage and oregano. On the other hand, Ahmed et al. (85) found no significant changes in live body weight and ADG in growing-finishing pigs fed a diet supplemented with medicinal plants such as pomegranate, *ginkgo biloba*, and licorice, compared with a control group. These studies highlight the variable effects of different plant extracts and herbal supplementation on growth performance in pigs, underscoring the importance of considering various factors when evaluating the efficacy of phytobiotics in swine nutrition. These factors include: species variation (different plant species have varying compositions of bioactive compounds, which can result in different effects on growth performance), plant parts and inherited characteristics (the specific plant parts used, such as leaves, stems, and roots), their inherent characteristics (chemical composition and nutrient content), age of plants (the age of plants at the time of harvest can affect the concentration and composition of bioactive compounds, and potentially influencing their impact on growth performance), timing of harvest (the timing of harvest in relation to the plant’s growth stage can influence the concentration and potency of bioactive compounds, which may affect their efficacy in promoting growth), and dosage variations (different dosages of herbs or their extracts can have varying effects on growth performance). Taking these factors into account is important in understanding the diverse outcomes observed in studies investigating the effects of herbs on animal growth performance (86–88).

## Conclusion

In recent times, the use of phytobiotics as natural growth promoters in the swine industry has gained increasing popularity. Various studies have demonstrated the potential of phytobiotics to exert anti-oxidative, antimicrobial, and anti-inflammatory effects. These findings suggest that phytochemicals could serve as a promising alternative to antibiotics in swine production, enhancing growth performance and health. While there is a general understanding of the effects of phytobiotics, there is still a lack of detailed information regarding their specific mechanisms of action, and the effectiveness of their implementation in practical settings. Therefore, further studies are necessary to investigate the modes of action of each type and dose of active compound in phytobiotics, as well as their potential interactions with other feed constituents. It is also important to assess the effects of phytobiotics throughout all phases of pig production to draw concrete conclusions regarding their use. By conducting more comprehensive research, we can gain a deeper understanding of phytobiotics and their potential benefits, allowing for their optimal utilization in swine nutrition and production. This will ultimately contribute to improved animal health and performance, as well as more sustainable and efficient swine farming practices.

## Author contributions

SP: Writing – review & editing, Writing – original draft, Resources. EK: Visualization, Writing – original draft, Writing – review & editing, Resources. JiC: Conceptualization, Writing – review & editing. MS: Conceptualization, Writing – review & editing. HD: Writing – original draft, Resources. SK: Conceptualization, Writing – review & editing, Resources. GK: Resources, Writing – original draft. JiK: Resources, Writing – original draft. SR: Resources, Writing – original draft. YC: Resources, Writing – original draft. JuK: Resources, Writing – original draft. JeC: Writing – review & editing, Conceptualization. HK: Conceptualization, Supervision, Validation, Writing – review & editing, Writing – original draft.

## Funding

The author(s) declare financial support was received for the research, authorship, and/or publication of this article. This work was supported by the National Research Foundation of Korea (NRF) funded by the Ministry of Education: (NRF-2021R1I1A3059910 and NRF-2019M3A9F3065227).

## Conflict of interest

The authors declare that the research was conducted in the absence of any commercial or financial relationships that could be construed as a potential conflict of interest.

## Publisher’s note

All claims expressed in this article are solely those of the authors and do not necessarily represent those of their affiliated organizations, or those of the publisher, the editors and the reviewers. Any product that may be evaluated in this article, or claim that may be made by its manufacturer, is not guaranteed or endorsed by the publisher.

## Figures and Tables

**Table:** 

AGPs	Antibiotic growth promoters
ADFI	Average daily feed intake
ADG	Average daily gain
BBA	Baicalin-aluminum complex
CAP	Capsicum oleoresin
FCR	Feed conversion ratio
GAR	Garlic botanical
HEM	Herbal extract mixture
JAM-A	Junctional adhesion molecule-A
LPS	Lipopolysaccharide
LW	Live weight
NC	Non-supplemented control
NF-kB	Nuclear factor-kB
PEO	Plant extraction oil
PWD	Post-weaning diarrhea
RNS	Reactive nitrogen radicals
ROS	Reactive oxygen species
RE	Rosemary extract
TCM	Traditional Chinese medicine
ZO	Zonula occludens

## References

[1] Das L Bhaumik E Raychaudhuri U Chakraborty R. Role of nutraceuticals in human health. J Food Sci Technol. (2012) 49:173–83. doi: 10.1007/s13197-011-0269-4, PMID: 23572839 PMC3550857

[2] Gupta RC Srivastava A Lall R. Nutraceuticals in veterinary medicine. Cham: Springer (2019).

[3] Filazi A Yurdakok-Dikmen B. Nutraceuticals in poultry health and disease In: Gupta R Srivastava A Lall R, editors. Nutraceuticals in veterinary medicine. Cham: Springer (2019). 661–72.

[4] Alagawany M Elnesr SS Farag MR Abd El-Hack ME Barkat RA Gabr AA. Potential role of important nutraceuticals in poultry performance and health-a comprehensive review. Res Vet Sci. (2021) 137:9–29. doi: 10.1016/j.rvsc.2021.04.009, PMID: 33915364

[5] Kim S Fan M Applegate T. Nonruminant nutrition symposium on natural Phytobiotics for health of young animals and poultry: mechanisms and application. J Anim Sci. (2008) 86:E138–9. doi: 10.2527/jas.2007-0769 18073287

[6] Grashorn M. Use of phytobiotics in broiler nutrition–an alternative to infeed antibiotics. J Anim Feed Sci. (2010) 19:338–47. doi: 10.22358/jafs/66297/2010

[7] Kikusato M. Phytobiotics to improve health and production of broiler chickens: functions beyond the antioxidant activity. Anim Biosci. (2021) 34:345. doi: 10.5713/ab.20.0842, PMID: 33705621 PMC7961201

[8] Serge Cyrille Houketchang N Herve KM. The use of plants as phytobiotics: a new challenge In: Marcos S-H Eva A-H Mariana P-T, editors. Phytochemicals in agriculture and food. Rijeka: IntechOpen (2023). Ch. 9.

[9] Li L Sun X Zhao D Dai H. Pharmacological applications and action mechanisms of phytochemicals as alternatives to antibiotics in pig production. Front Immunol. (2021) 12:798553. doi: 10.3389/fimmu.2021.798553, PMID: 34956234 PMC8695855

[10] Mandey JS Sompie FN. Phytogenic feed additives as an alternative to antibiotic growth promoters in poultry nutrition In: Babinszky L Oliveira J Mauro Santos E, editors. Advanced studies in the 21st century animal nutrition. London: IntechOpen Limited (2021). 19.

[11] Dialoke N Onimisi P Afolayan M. Performance, blood parameters and economic indices of broiler chickens fed graded levels of chestnut (Castenea sativa) phytobiotics as replacement for antibiotics growth promoters. Niger J Anim Prod. (2020) 47:161–70. doi: 10.51791/njap.v47i2.123

[12] Suryanarayana M Durga S. Role of phytogenic feed additives in swine production-a review. Int. J. Environ. Agric. Biotech. (2018) 3:264375. doi: 10.22161/ijeab/3.3.46

[13] Windisch W Schedle K Plitzner C Kroismayr A. Use of phytogenic products as feed additives for swine and poultry. J Anim Sci. (2008) 86:E140–8. doi: 10.2527/jas.2007-0459 18073277

[14] Lin ZN Ye L Li ZW Huang XS Lu Z Yang YQ. Chinese herb feed additives improved the growth performance, meat quality, and nutrient digestibility parameters of pigs. Anim Model Exp Med. (2020) 3:47–54. doi: 10.1002/ame2.12104, PMID: 32318659 PMC7167239

[15] Krauze M. Phytobiotics, a natural growth promoter for poultry In: Babinszky L Oliveira J Mauro Santos E, editors. Advanced studies in the 21st century animal nutrition. London: IntechOpen Limited (2021)

[16] Lillehoj H Liu Y Calsamiglia S Fernandez-Miyakawa ME Chi F Cravens RL. Phytochemicals as antibiotic alternatives to promote growth and enhance host health. Vet Res. (2018) 49:76. doi: 10.1186/s13567-018-0562-6 30060764 PMC6066919

[17] Pashtetsky V Ostapchuk P Kuevda T Zubochenko D Yemelianov S Uppe V, editors. Use of phytobiotics in animal husbandry and poultry. E3S Web of Conferences; (2020). EDP Sciences.

[18] Vidanarachchi JK Mikkelsen L Sims I Iji P Choct M. Phytobiotics: alternatives to antibiotic growth promoters in monogastric animal feeds. Recent Advances in Animal Nutrition in Australia, Vol. 15, pp. 131–144 (2005). Available at: https://hdl.handle.net/1959.11/4186

[19] Deng Z-Y Zhang J-W Li J Fan Y-W Cao S-W Huang R-l. Effect of polysaccharides of cassiae seeds on the intestinal microflora of piglets. Asia Pac J Clin Nutr. (2007) 16:143–7. 17392093

[20] Wang L Huang Y Yin G Wang J Wang P Chen ZY. Antimicrobial activities of Asian ginseng, American ginseng, and notoginseng. Phytother Res. (2020) 34:1226–36. doi: 10.1002/ptr.6605, PMID: 31885119

[21] Weber WM Hunsaker LA Abcouwer SF Deck LM Vander Jagt DL. Anti-oxidant activities of curcumin and related enones. Bioorg Med Chem. (2005) 13:3811–20. doi: 10.1016/j.bmc.2005.03.035, PMID: 15863007

[22] Esatbeyoglu T Huebbe P Ernst IM Chin D Wagner AE Rimbach G. Curcumin—from molecule to biological function. Angew Chem Int Ed. (2012) 51:5308–32. doi: 10.1002/anie.201107724, PMID: 22566109

[23] Spanier G Xu H Xia N Tobias S Deng S Wojnowski L. Resveratrol reduces endothelial oxidative stress by modulating the gene expression of superoxide dismutase 1 (Sod1), glutathione peroxidase 1 (Gpx1) and Nadph oxidase subunit (Nox4). J Physiol Pharmacol. (2009) 60:111–6. 20083859

[24] Wei A Shibamoto T. Antioxidant activities and volatile constituents of various essential oils. J Agric Food Chem. (2007) 55:1737–42. doi: 10.1021/jf062959x, PMID: 17295511

[25] Mohammadi Gheisar M Kim IH. Phytobiotics in poultry and swine nutrition–a review. Ital J Anim Sci. (2018) 17:92–9. doi: 10.1080/1828051X.2017.1350120

[26] Dávila-Ramírez JL Munguía-Acosta LL Morales-Coronado JG García-Salinas AD González-Ríos H Celaya-Michel H. Addition of a mixture of plant extracts to diets for growing-finishing pigs on growth performance, blood metabolites, carcass traits, organ weight as a percentage of live weight, quality and sensorial analysis of meat. Animals. (2020) 10:1229. doi: 10.3390/ani10071229, PMID: 32698311 PMC7401503

[27] Janz J Morel P Wilkinson B Purchas R. Preliminary investigation of the effects of low-level dietary inclusion of fragrant essential oils and oleoresins on pig performance and pork quality. Meat Sci. (2007) 75:350–5. doi: 10.1016/j.meatsci.2006.06.027, PMID: 22063669

[28] Al-Kassie GA. Influence of two plant extracts derived from thyme and cinnamon on broiler performance. Pak Vet J. (2009) 29:169–73.

[29] Su G Zhou X Wang Y Chen D Chen G Li Y. Dietary supplementation of plant essential oil improves growth performance, intestinal morphology and health in weaned pigs. J Anim Physiol Anim Nutr. (2020) 104:579–89. doi: 10.1111/jpn.13271 31854008

[30] Liu Y Song M Che T Lee J Bravo D Maddox C. Dietary plant extracts modulate gene expression profiles in Ileal mucosa of weaned pigs after an *Escherichia Coli* infection. J Anim Sci. (2014) 92:2050–62. doi: 10.2527/jas.2013-6422, PMID: 24663182

[31] Ahmed S Hossain M Kim G Hwang J Ji H Yang C. Effects of resveratrol and essential oils on growth performance, immunity, digestibility and fecal microbial shedding in challenged piglets. Asian Australas J Anim Sci. (2013) 26:683. doi: 10.5713/ajas.2012.12683, PMID: 25049839 PMC4093338

[32] Li P Piao X Ru Y Han X Xue L Zhang H. Effects of adding essential oil to the diet of weaned pigs on performance, nutrient utilization, immune response and intestinal health. Asian Australas J Anim Sci. (2012) 25:1617. doi: 10.5713/ajas.2012.12292, PMID: 25049525 PMC4093040

[33] Manzanilla E Perez J Martin M Kamel C Baucells F Gasa J. Effect of plant extracts and formic acid on the intestinal equilibrium of early-weaned pigs. J Anim Sci. (2004) 82:3210–8. doi: 10.2527/2004.82113210x, PMID: 15542467

[34] Qu H Huang Y Shi Y Liu Y Wu S Bao W. Effect of bamboo vinegar powder as an antibiotic alternative on the digesta bacteria communities of finishing pigs. Can J Microbiol. (2018) 64:732–43. doi: 10.1139/cjm-2018-0058, PMID: 29775545

[35] Satora M Magdziarz M Rząsa A Rypuła K Płoneczka-Janeczko K. Insight into the intestinal microbiome of farrowing sows following the administration of garlic (*Allium Sativum*) extract and probiotic bacteria cultures under farming conditions. BMC Vet Res. (2020) 16:442. doi: 10.1186/s12917-020-02659-y 33187511 PMC7666521

[36] Vasconcelos N Croda J Simionatto S. Antibacterial mechanisms of cinnamon and its constituents: a review. Microb Pathog. (2018) 120:198–203. doi: 10.1016/j.micpath.2018.04.036, PMID: 29702210

[37] Girard M Hu D Pradervand N Neuenschwander S Bee G. Chestnut extract but not sodium salicylate decreases the severity of diarrhea and Enterotoxigenic *Escherichia Coli* F4 shedding in artificially infected piglets. PLoS One. (2020) 15:e0214267. doi: 10.1371/journal.pone.0214267, PMID: 32106264 PMC7046202

[38] Fu S Zhuang F Guo L Qiu Y Xiong J Ye C. Effect of Baicalin-aluminum complexes on fecal microbiome in piglets. Int J Mol Sci. (2019) 20:2390. doi: 10.3390/ijms20102390, PMID: 31091773 PMC6566245

[39] Yang M Yin Y Wang F Bao X Long L Tan B. Effects of dietary rosemary extract supplementation on growth performance, nutrient digestibility, antioxidant capacity, intestinal morphology, and microbiota of weaning pigs. J Anim Sci. (2021) 99:skab237. doi: 10.1093/jas/skab237 34370023 PMC8420665

[40] Yan L Meng Q Kim I. The effect of an herb extract mixture on growth performance, nutrient digestibility, blood characteristics and fecal noxious gas content in growing pigs. Livest Sci. (2011) 141:143–7. doi: 10.1016/j.livsci.2011.05.011

[41] Marcin A Lauková A Mati R. Comparison of the effects of enterococcus faecium and aromatic oils from sage and oregano on growth performance and diarrhoeal diseases of weaned pigs. Biologia. (2006) 61:789–95. doi: 10.2478/s11756-006-0159-9

[42] Dundar E Olgun EG Isiksoy S Kurkcuoglu M Baser KHC Bal C. The effects of intra-rectal and intra-peritoneal application of *Origanum Onites* L. essential oil on 2, 4, 6-trinitrobenzenesulfonic acid-induced colitis in the rat. Exp Toxicol Pathol. (2008) 59:399–408. doi: 10.1016/j.etp.2007.11.009, PMID: 18222658

[43] Ulrikh EV Khaliullin RS Ganieva IA Izhmulkina E Arzjutov M. The content of biologically active substances in phytobiotics used for agricultural animals and poultry. Int J Eng Technol (UAE). (2018) 7:445–9. doi: 10.14419/ijet.v7i3.14.17040

[44] Huyghebaert G Ducatelle R Van Immerseel F. An update on alternatives to antimicrobial growth promoters for broilers. Vet J. (2011) 187:182–8. doi: 10.1016/j.tvjl.2010.03.003, PMID: 20382054

[45] Fresno Rueda A Samuel R St-Pierre B. Investigating the effects of a phytobiotics-based product on the fecal bacterial microbiome of weaned pigs. Animals. (2021) 11:1950. doi: 10.3390/ani11071950, PMID: 34208843 PMC8300416

[46] Ducatelle R Goossens E De Meyer F Eeckhaut V Antonissen G Haesebrouck F. Biomarkers for monitoring intestinal health in poultry: present status and future perspectives. Vet Res. (2018) 49:43. doi: 10.1186/s13567-018-0538-6 29739469 PMC5941335

[47] Castillo M Martín-Orúe S Roca M Manzanilla E Badiola I Perez J. The response of gastrointestinal microbiota to Avilamycin, butyrate, and plant extracts in early-weaned pigs. J Anim Sci. (2006) 84:2725–34. doi: 10.2527/jas.2004-556, PMID: 16971574

[48] Li Z Lin Z Lu Z Feng Z Chen Q Deng S. Coix seed improves growth performance and productivity in post-weaning pigs by reducing gut Ph and modulating gut microbiota. AMB Express. (2019) 9:115. doi: 10.1186/s13568-019-0828-z 31338616 PMC6650524

[49] Iqbal Y Cottrell JJ Suleria HA Dunshea FR. Gut microbiota-polyphenol interactions in chicken: a review. Animals. (2020) 10:1391. doi: 10.3390/ani10081391, PMID: 32796556 PMC7460082

[50] Suzuki T. Regulation of the intestinal barrier by nutrients: the role of tight junctions. Anim Sci J. (2020) 91:e13357. doi: 10.1111/asj.13357, PMID: 32219956 PMC7187240

[51] Mayangsari Y Suzuki T. Resveratrol ameliorates intestinal barrier defects and inflammation in colitic mice and intestinal cells. J Agric Food Chem. (2018) 66:12666–74. doi: 10.1021/acs.jafc.8b04138, PMID: 30426751

[52] Azuma T Shigeshiro M Kodama M Tanabe S Suzuki T. Supplemental naringenin prevents intestinal barrier defects and inflammation in colitic mice. J Nutr. (2013) 143:827–34. doi: 10.3945/jn.113.174508, PMID: 23596159

[53] Burt S. Essential oils: their antibacterial properties and potential applications in foods—a review. Int J Food Microbiol. (2004) 94:223–53. doi: 10.1016/j.ijfoodmicro.2004.03.022, PMID: 15246235

[54] Panghal M Kaushal V Yadav JP. In vitro antimicrobial activity of ten medicinal plants against clinical isolates of oral cancer cases. Ann Clin Microbiol Antimicrob. (2011) 10:21. doi: 10.1186/1476-0711-10-21 21599889 PMC3121585

[55] Si W Gong J Tsao R Zhou T Yu H Poppe C. Antimicrobial activity of essential oils and structurally related synthetic food additives towards selected pathogenic and beneficial gut bacteria. J Appl Microbiol. (2006) 100:296–305. doi: 10.1111/j.1365-2672.2005.02789.x, PMID: 16430506

[56] Yang C Chowdhury MK Hou Y Gong J. Phytogenic compounds as alternatives to in-feed antibiotics: potentials and challenges in application. Pathogens. (2015) 4:137–56. doi: 10.3390/pathogens4010137, PMID: 25806623 PMC4384076

[57] Salehi B Mishra AP Shukla I Sharifi-Rad M Contreras MM Segura-Carretero A. Thymol, thyme, and other plant sources: health and potential uses. Phytother Res. (2018) 32:1688–706. doi: 10.1002/ptr.6109, PMID: 29785774

[58] Valenzuela-Grijalva NV Pinelli-Saavedra A Muhlia-Almazan A Domínguez-Díaz D González-Ríos H. Dietary inclusion effects of phytochemicals as growth promoters in animal production. J Anim Sci Technol. (2017) 59:8. doi: 10.1186/s40781-017-0133-9 28428891 PMC5392986

[59] Di Pasqua R Betts G Hoskins N Edwards M Ercolini D Mauriello G. Membrane toxicity of antimicrobial compounds from essential oils. J Agric Food Chem. (2007) 55:4863–70. doi: 10.1021/jf0636465, PMID: 17497876

[60] Gill A Holley R. Inhibition of membrane bound atpases of Escherichia Coli and *Listeria Monocytogenes* by plant oil aromatics. Int J Food Microbiol. (2006) 111:170–4. doi: 10.1016/j.ijfoodmicro.2006.04.046, PMID: 16828188

[61] Zhou F Ji B Zhang H Jiang H Yang Z Li J. Synergistic effect of thymol and carvacrol combined with chelators and organic acids against *Salmonella Typhimurium*. J Food Prot. (2007) 70:1704–9. doi: 10.4315/0362-028X-70.7.1704, PMID: 17685346

[62] Omonijo FA Ni L Gong J Wang Q Lahaye L Yang C. Essential oils as alternatives to antibiotics in swine production. Anim Nutr. (2018) 4:126–36. doi: 10.1016/j.aninu.2017.09.001, PMID: 30140752 PMC6104524

[63] Chang SY Song MH Lee JH Oh HJ Kim YJ An JW. Phytogenic feed additives alleviate pathogenic *Escherichia Coli*-induced intestinal damage through improving barrier integrity and inhibiting inflammation in weaned pigs. J Anim Sci Biotechnol. (2022) 13:107. doi: 10.1186/s40104-022-00750-y 36050784 PMC9438252

[64] Huang C Wang Y He X Jiao N Zhang X Qiu K. The involvement of Nf-Κb/P38 pathways in Scutellaria baicalensis extracts attenuating of *Escherichia Coli* K88-induced acute intestinal injury in weaned piglets. Br J Nutr. (2019) 122:152–61. doi: 10.1017/S0007114519000928, PMID: 31006408

[65] Cruzen S Baumgard L Gabler N Pearce S Lonergan S. Temporal proteomic response to acute heat stress in the porcine muscle sarcoplasm. J Anim Sci. (2017) 95:3961–71. doi: 10.2527/jas2017.1375, PMID: 28992025

[66] Hao Y Xing M Gu X. Research progress on oxidative stress and its nutritional regulation strategies in pigs. Animals. (2021) 11:1384. doi: 10.3390/ani11051384, PMID: 34068057 PMC8152462

[67] Tsai W-H Yang C-C Li P-C Chen W-C Chien C-T. Therapeutic potential of traditional Chinese medicine on inflammatory diseases. J Tradit Complement Med. (2013) 3:142–51. doi: 10.4103/2225-4110.114898, PMID: 24716170 PMC3924991

[68] Shingnaisui K Dey T Manna P Kalita J. Therapeutic potentials of *Houttuynia Cordata* Thunb. against inflammation and oxidative stress: a review. J Ethnopharmacol. (2018) 220:35–43. doi: 10.1016/j.jep.2018.03.038, PMID: 29605674 PMC7127360

[69] Shaw P Chattopadhyay A. Nrf2–are signaling in cellular protection: mechanism of action and the regulatory mechanisms. J Cell Physiol. (2020) 235:3119–30. doi: 10.1002/jcp.29219, PMID: 31549397 PMC13482834

[70] Niture SK Kaspar JW Shen J Jaiswal AK. Nrf2 signaling and cell survival. Toxicol Appl Pharmacol. (2010) 244:37–42. doi: 10.1016/j.taap.2009.06.009, PMID: 19538984 PMC2837794

[71] Kaltschmidt C Greiner JF Kaltschmidt B. The transcription factor Nf-Κb in stem cells and development. Cells. (2021) 10:2042. doi: 10.3390/cells10082042, PMID: 34440811 PMC8391683

[72] Wang X Wang Y Mao Y Hu A Xu T Yang Y. The beneficial effects of traditional Chinese medicine on antioxidative status and inflammatory cytokines expression in the liver of piglets. Front Vet Sci. (2022) 9:937745. doi: 10.3389/fvets.2022.1063573, PMID: 36213414 PMC9539681

[73] Park MH Hong JT. Roles of Nf-Κb in cancer and inflammatory diseases and their therapeutic approaches. Cells. (2016) 5:15. doi: 10.3390/cells5020015, PMID: 27043634 PMC4931664

[74] Liu T Zhang L Joo D Sun S-C. Nf-Κb signaling in inflammation. Signal Transduct Target Ther. (2017) 2:1–9. doi: 10.1038/sigtrans.2017.23 PMC5661633 29158945

[75] Trachootham D Lu W Ogasawara MA Valle NR-D Huang P. Redox regulation of cell survival. Antioxid Redox Signal. (2008) 10:1343–74. doi: 10.1089/ars.2007.1957, PMID: 18522489 PMC2932530

[76] Cao S Shen Z Wang C Zhang Q Hong Q He Y. Resveratrol improves intestinal barrier function, alleviates mitochondrial dysfunction and induces mitophagy in diquat challenged piglets 1. Food Funct. (2019) 10:344–54. doi: 10.1039/C8FO02091D, PMID: 30601541

[77] Bartoš P Dolan A Smutný L Šístková M Celjak I Šoch M. Effects of phytogenic feed additives on growth performance and on ammonia and greenhouse gases emissions in growing-finishing pigs. Anim Feed Sci Technol. (2016) 212:143–8. doi: 10.1016/j.anifeedsci.2015.11.003

[78] Jugl-Chizzola M Ungerhofer E Gabler C Hagmüller W Chizzola R Zitterl-Eglseer K. Testing of the palatability of *Thymus Vulgaris* L. and *Origanum Vulgare* L. as flavouring feed additive for weaner pigs on the basis of a choice experiment. Berl Munch Tierarztl Wochenschr. (2006) 119:238–43. PMID: 16729471

[79] Schöne F Vetter A Hartung H Bergmann H Biertümpfel A Richter G. Effects of essential oils from fennel (Foeniculi Aetheroleum) and caraway (Carvi Aetheroleum) in pigs. J Anim Physiol Anim Nutr. (2006) 90:500–10. doi: 10.1111/j.1439-0396.2006.00632.x, PMID: 17083431

[80] Zhai H Liu H Wang S Wu J Kluenter A-M. Potential of essential oils for poultry and pigs. Anim Nutr. (2018) 4:179–86. doi: 10.1016/j.aninu.2018.01.005, PMID: 30140757 PMC6103468

[81] Costa L Luciano F Miyada VS Gois F. Herbal extracts and organic acids as natural feed additives in pig diets. South Afr J Anim Sci. (2013) 43:181–93.

[82] Jamroz D Wiliczkiewicz A Wertelecki T Orda J Skorupińska J. Use of active substances of plant origin in chicken diets based on maize and locally grown cereals. Br Poult Sci. (2005) 46:485–93. doi: 10.1080/00071660500191056 16268107

[83] Jang I Ko Y Kang S Lee C. Effect of a commercial essential oil on growth performance, digestive enzyme activity and intestinal microflora population in broiler chickens. Anim Feed Sci Technol. (2007) 134:304–15. doi: 10.1016/j.anifeedsci.2006.06.009

[84] Diaz-Sanchez S D'Souza D Biswas D Hanning I. Botanical alternatives to antibiotics for use in organic poultry production. Poult Sci. (2015) 94:1419–30. doi: 10.3382/ps/pev014, PMID: 25743421

[85] Ahmed ST Mun H-S Islam MM Ko S-Y Yang C-J. Effects of dietary natural and fermented herb combination on growth performance, carcass traits and meat quality in grower-finisher pigs. Meat Sci. (2016) 122:7–15. doi: 10.1016/j.meatsci.2016.07.016, PMID: 27468138

[86] Hashemi S Davoodi H. Phytogenics as new class of feed additive in poultry industry. J Anim Vet Adv. (2010) 9:2295–304. doi: 10.3923/javaa.2010.2295.2304

[87] Zhang G Lee Y Wang Z-Y Wang Y. Synthesis and bioactivities of plant-derived biomolecules. Front Plant Sci. (2022) 13:949057. doi: 10.3389/fpls.2022.1077403, PMID: 35812945 PMC9270015

[88] Wenk C. Herbs and botanicals as feed additives in Monogastric animals. Asian Australas J Anim Sci. (2003) 16:282–9. doi: 10.5713/ajas.2003.282

